# Concentration and distribution of sialic acid in human milk and its correlation with dietary intake

**DOI:** 10.3389/fnut.2022.929661

**Published:** 2022-08-04

**Authors:** Qiaoling Xie, Yuhan Xu, Wei Zhang, Meizhen Zhu, Xinyue Wang, Jiale Huang, Yingying Zhuang, Hui Lan, Xiaoxuan Chen, Dongbei Guo, Hongwei Li

**Affiliations:** ^1^School of Public Health, Xiamen University, Xiamen, China; ^2^Department of Clinical Nutrition, School of Medicine, Ruijin Hospital Affiliated to Shanghai Jiao Tong University, Shanghai, China; ^3^Department of Clinical Nutrition, The First Affiliated Hospital of Nanchang University, Nanchang, China; ^4^Department of Clinical Nutrition, Xiamen Humanity Hospital, Xiemen, China

**Keywords:** human milk, diet, sialic acid, distribution, content

## Abstract

**Purpose:**

This study evaluates the content, distribution, and changing trend of sialic acid in human milk and the correlation between dietary intake of sialic acid and that in human milk.

**Methods:**

The study included 33 mothers of full-term and exclusively breastfed infants. At least 2 ml of milk was collected on the 3rd, 8th, 30th, and 90th day after delivery, and 24-h diet recalls of the lactating mothers were obtained each time. The correlation of human milk sialic acid concentration with lactating women's dietary sialic acid intake during lactation was analyzed by statistical analysis software SPSS.

**Results:**

The average concentration of sialic acid in colostrum, transition, and 1 and 3 months were 1,670.74 ± 94.53, 1,272.19 ± 128.74, 541.64 ± 55.2, and 297.65 ± 20.78 mg/L, respectively. The total sialic acid concentration in colostrum was about 5.6 times higher than that at 3 months (*P* < 0.001). The average dietary sialic acid intake of lactating mothers on the 2nd, 7th, 30th, and 90th day after delivery were 106.06 ± 7.51, 127.64 ± 8.61, 120.34 ± 10.21, and 95.40 ± 6.34 mg/day, respectively. The intake of sialic acid was relatively high on the 7th day, and there was no significant difference in dietary intake of sialic acid on different days (*P* < 0.05). In addition, there was no correlation between the intake of dietary sialic acid and the content of total sialic acid and various forms of sialic acid in milk (*P* < 0.05).

**Conclusion:**

During the lactation period, the distribution of sialic acid in breast milk is relatively stable and its content fluctuates greatly, which may not be affected by the mother's diet, but mainly depends on the self-regulation oft physiological needs.

## Introduction

Sialic acid is widespread in human tissues, especially the central nervous system. Most of the sialic acid in brain tissue binds to gangliosides, accounting for about 65% of the total sialic acid content of the brain; about 32% is bound to glycoproteins, and the rest exists in the free form (1). The high content of sialic acid is an important material basis of nervous system function, meaning that it may play a unique structural and functional role in the nervous system. Interventions with sialic acid can have long-term irreversible effects on the brain during critical periods of brain development.

Wang (2) fed piglets with sialic acid-supplemented formula, and the eight-arm maze test was conducted to evaluate their learning and memory ability. The results showed that sialic acid affected the brains of piglets and could significantly improve their learning and memory ability. Morgan and Winick's study (3) confirmed that increasing exogenous sialic acid intake could promote ganglioside sialic acid production in the brain of young rats and improve learning ability, and these changes will persist into adulthood. Moreover, some studies had found that *N*-acetylneuraminic acid supplementation to the mother rats during pregnancy could enhance the brain Neu5Ac and promote the cognitive development of the offspring but had no such effect with rats that missed the period of rapid brain development (4, 5). Thus, these findings suggest that sialic acid is vital for brain growth and development, but only increased exogenous sialic acid intake early in life affects its content in brain tissue.

All mammals, including humans, can synthesize endogenous sialic acid. However, endogenous synthesis is limited for newborns because the liver and other organs are not yet matured. Hence, infants need adequate exogenous sialic acid to meet rapid brain development needs. Human milk is the primary exogenous source of sialic acid, which mainly exists in combined form in oligosaccharides, glycoproteins, and glycolipids in human milk. Studies have shown that sialic acid is abundant in colostrum and tends to decrease with prolonged lactation (2, 6). Changes in the content of sialic acid in human milk can be affected by various factors, and whether they are affected by dietary factors is unknown. There is relatively little research on the composition, distribution, and related influencing factors of sialic acid in human milk. We analyzed the distribution, content, and trend of sialic acid in human milk, its changing trend with lactation continuation, the correlation between dietary sialic acid intake and sialic acid in human milk, and discussed its nutritional significance.

## Materials and methods

### Dietary sialic acid survey

The study included 33 mothers of full-term and exclusively breastfed infants from the Maternal and Child Care Service Center in Xiamen, China. The household survey was conducted on the 3rd, 8th, 30th, and 90th postnatal days, and a 24-h dietary recall of all foods and drinks consumed was obtained on the survey days. We use a questionnaire combined with a special food quantity reference measurer for dietary survey, including commonly used food pictures, quantitative standard molds and common food weight tables to assist respondents in recall, qualitative and quantitative analysis. Record the variety, brand name, texture and processing of food. Fill in in grams, if other units, convert to grams, and accurately calculate the type and weight of food. The investigation was conducted by specially trained investigators using the same protocol. In addition, follow-up data collection for quality control was conducted through home visits or telephone calls to verify and supplement the data. The dietary composition of sialic acid was determined in the laboratory, and the intake of dietary sialic acid in lactating women was estimated. The study was approved by the Medical Ethics Committee (Ethics No: XDYX2020008), and all the mothers gave written informed consent before inclusion in the study.

### Basic information of subjects

Almost all the mothers surveyed were the first-born, mainly permanent residents of Xiamen, basically Han nationality, with a good level of education. The average age of all mothers was about 27 years old, and the average height was 160cm. Their body weight decreased with lactation, but the average body mass index (BMI) was within normal range. All the nursing mothers are healthy non-smokers and have no family history of genetic diseases. They don't drink or take drugs in puerperium, and they start to exercise moderately after the month.

### Human milk collection

Breast milk samples (2–10 ml) were collected using a breast pump at approximately the same time of the morning (09:00–11:00) on 4 occasions: 3rd (colostrum), 8th (transition milk), 30th (mature milk), and 90th (mature milk) days after birth. All milk samples were stored in clean bottles in a −20°C refrigerator until analyzed.

### Preparation and determination of human milk samples

#### Preparation and handling of human milk samples

First, 500 μl of milk was accurately aspirated in a centrifuge tube, and an equal volume of 10% trichloroacetic acid solution was added to precipitate the protein. The solution was thoroughly mixed, then placed in an ice bath for 10 min, centrifuged at 4°C, 3,000 ( × g) for 30 min, and the supernatant was collected. Secondly, 500 μl cold 5% trichloroacetic acid was added to the precipitate, mixed well, centrifuged for 30 min at 4°C, 3,000 rpm, and the supernatant was mixed with the previous one. Next, 500 μl of supernatant was taken in a centrifuge tube and filtered through a 0.22 μm membrane for free sialic acid detection. The remaining supernatant was added with an equal volume of 0.1 mol/L trifluoroacetic acid, hydrolyzed at 80°C for 30 min, and filtered through a 0.22 μm filter membrane after cooling. Taking the filtrate for detection, the sum of free sialic acid and oligosaccharide-bound sialic acid in human milk can be obtained. Subtracting free sialic acid from this part of the result is oligosaccharide-bound sialic acid. In addition, 2 ml (0.05 mol/L) sulfuric acid was added to the precipitated protein, hydrolyzed at 80°C for 120 min. After cooling, it was filtered through a 0.22 μm filter membrane, and the liquid was taken for protein-binding sialic acid detection (7).

Although a small part of sialic acid also exists in the form of glycolipids (gangliosides), its proportion in the total sialic acid content of human milk is very low (<0.5%) (8); therefore, it was not detected separately in this study (included in free sialic acid).

#### Chromatographic detection conditions

Chromatographic column: waters C18 (2.5 μm, 2.1 × 150 mm), protective column: waters C18 (2.5 μm, 2.1 × 20 mm), column temperature 30°C; The excitation wavelength of the fluorescence detector was 373 nm, and the emission wavelength was 448 nm; The mobile phase was methanol-acetonitrile-ultrapure water (2.5:3.5:94); the flow rate was 0.3 ml/min; the injection volume was 10 μl.

Dimethyl balenine (DMB) derivatization solution: 8 mM DMB, 1.5 M glacial acetic acid, 0.25 M sodium hyposulfifite, 0.25 M sodium sulfifite, and 0.8 mM 2-mercaptoethanol.

Derivatization conditions: 10 μl DMB derivatization solution was added to 90 μl of filtered samples or standards, derivatized at 50°C for 150 min in the dark, cooled to room temperature, and analyzed by liquid spectrometry (4).

Repeatability test: the colostrum of the same person was measured for six repeated samples, 500 μl each. After injection according to the sample treatment method, the peak area was determined to be 7,456, 7,436, 7,320, 7,459, 7,489, and 7468 respectively, and the calculated RSD was 0.8%, indicating that the detection method had good repeatability.

### Statistical analysis

The one-way ANOVA in a simple linear model was used to evaluate the variation among the four lactation periods. Bonferroni correction and repeated measures ANOVA were used to analyze the dynamic changes of sialic acid contents of dietary intake and in human milk of nursing mothers during lactation. Shapiro–Wilk test and Spearman's correlation coefficient were used to determine the correlation between sialic acid content in the mothers' dietary intake and the milk secreted. The significance level was set at *P* = 0.05.

## Results

### Sialic acid content and changing trend in human milk

Table 1 shows that colostrum had the highest (1,670.74 mg/L) sialic acid content. As the lactation period increased, the sialic acid content in human milk decreased rapidly. The content of transition milk decreased to 1,272.19 mg/L, and mature milk content at 1 month and 3 months was 541.64 and 297.65 mg/L, respectively. The total sialic acid concentration in colostrum was about 5.6 times that of mature milk at 3 months (*P* < 0.001), with 16%−19% of that in colostrum. The concentration of total sialic acid in human milk was negatively correlated with postpartum days (*r* = 0.834 *P* < 0.001). The difference in total sialic acid concentration among different time points was statistically significant (*P* < 0.05). This concentration difference was also reflected in oligosaccharide-bound, protein-bound, and free sialic acid at the four-time points during lactation (*P* < 0.05).

**Table 1:** The concentration of sialic acid in human milk at 4-time points during lactation (mg/L, mean ± SE, *n* = 33).

**Distribution of sialic acid**	**Colostrum**	**Transition**	**Mature, 30th day**	**Mature, 90th day**
Free SA	51.58 ± 2.92	38.08 ± 3.85^*^	16.52 ± 1.78^*^	9.26 ± 0.60^*^
Oligosaccharide-bound SA	1,210.71 ± 68.13	917.28 ± 92.40^*^	395.71 ± 40.75^*^	213.57 ± 15.23^*^
Protein-bound SA	408.45 ± 25.16	316.82 ± 33.28^*^	129.41 ± 12.89^*^	74.82 ± 5.13^*^
Total SA	1,670.74 ± 94.53	1,272.19 ± 128.74^*^	541.64 ± 55.20^*^	297.65 ± 20.78^*^

* Indicates that there is a statistical difference compared with the previous time point, P < 0.05.

The total sialic acid concentration and that in each fraction showed a decreasing trend. After the repeatability test, it was found to change significantly with continued lactation (*F* = 200.796, *P* < 0.001). The concentration of sialic acid in milk differed between species (*F* = 174.97, *P* < 0.001). The changing trend of sialic acid in different forms differed with time (*F* = 119.1, *P* < 0.001), and the decreasing trend of oligosaccharide-bound sialic acid was more obvious than that of protein-bound and free sialic acid.

### Distribution of sialic acid in human milk

**Figure 1:**
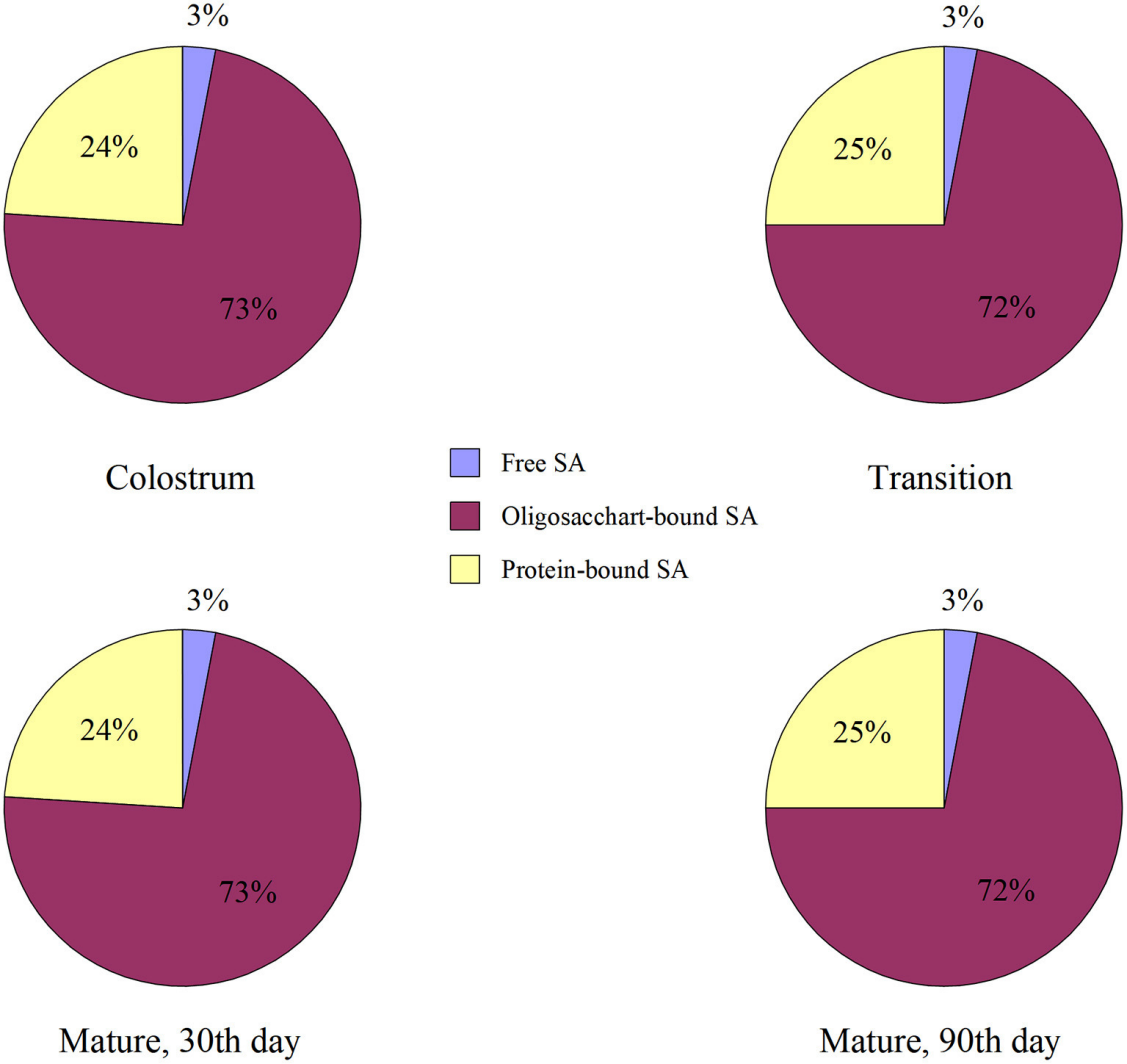
Distribution of sialic acids in human milk in different lactation. The milk samples of 33 mothers were collected on the 3rd, 8th, 30th and 90th day during lactation to detect the content of sialic acid. The results showed the proportion of different forms of sialic acid at different time points. Colostrum, 3rd; Transition, 8th; The results are expressed as percentage (%); *n* = 33.

**Figure 2:**
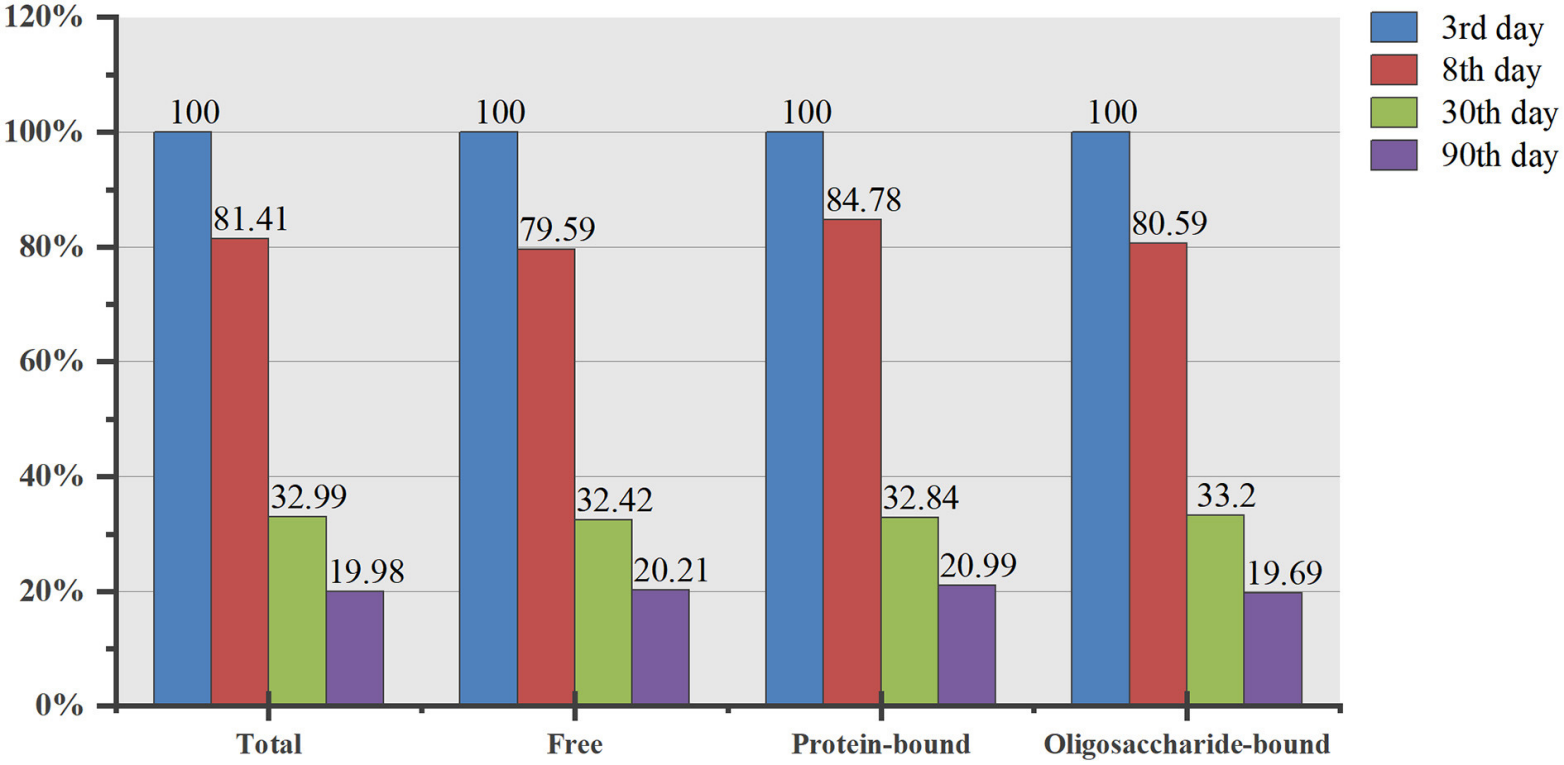
Variation trend of total and each fraction of sialic acid compared with colostrum. The results are expressed as percentage (%); *n* = 33.

Most of the sialic acid in human milk (67.6%−76.0%) was bound to oligosaccharides, 20.9%−29.4% combined with protein, and 2.2%−3.6% was free sialic acid. Figure 1 shows that no matter the lactation stage, the proportion was highest in oligosaccharide-bound sialic acid, and the ratio of the three components was unchanged. As shown in Figure 2, the contents of free, oligosaccharide-bound, protein-bound, and total sialic acid in the colostrum are used as references. The content of total sialic acid in transitional milk, one-month mature milk, and 3-month mature milk was 81.41%, 32.99%, and 19.98% of colostrum; the corresponding content of free sialic acid was 79.59%, 32.42%, and 20.21%; the content bound with oligosaccharides was 80.59%, 33.20% and 19.69%; and that bound to protein was 84.78%, 32.84%, and 20.99%. With the prolonged lactation period, the total sialic acid and that in other forms decreased significantly compared with that in the colostrum, and the proportion of decrease was similar.

### Correlation analysis between dietary sialic acid intake and sialic acid content in milk

Table 2 shows the average dietary Neu5Ac intake of lactating mothers on the 2nd, 7th, 30th, and 90th days after delivery. The average dietary intake of total sialic acid of lactating mothers was the highest on the 7th day after delivery (127.6 ± 48.61 mg/day) and decreased by about 25% in the 3rd month (*P* < 0.05).

**Table 2:** The concentration of sialic acid in human milk at 4-time points during lactation (mg/L, mean ± SE, *n* = 33).

	**Dietary total**	**Total SA**	**Total SA content**	**Oligosaccharide-**	**Protein-bound SA**	**Free SA content**
	**SA intake**	**concentration in**	**in milk**	**bound SA content**	**content in**	**in milk**
	**(mg/day)**	**milk (mg/L)**	**(mg/day)**	**in milk (mg/L)**	**milk (mg/L)**	**in milk (mg/L)**
2nd day	106.06 ± 7.51	1,670.74 ± 94.53	167.07 ± 9.45	1,210.71 ± 68.13	408.45 ± 25.16	51.58 ± 2.92
*r* ^*^		0.127	0.127	0.102	0.186	0.135
*P*-value		0.480	0.480	0.574	0.300	0.453
7th day	127.64 ± 8.61^a^	1,272.19 ± 128.74	636.09 ± 64.37	917.28 ± 92.4	316.82 ± 33.28	38.08 ± 3.85
*r* ^*^		0.152	0.152	0.152	0.143	0.141
*P*-value		0.398	0.398	0.399	0.427	0.435
30th day	120.34 ± 10.21^a^	541.64 ± 55.2	352.07 ± 35.88	395.71 ± 40.75	129.41 ± 12.89	16.52 ± 1.78
*r* ^*^		−0.280	−0.280	−0.287	−0.244	−0.261
*P*-value		0.114	0.114	0.105	0.171	0.143
90th day	95.40 ± 6.34	297.65 ± 20.78	267.89 ± 18.7	213.57 ± 15.23	74.82 ± 5.13	9.26 ± 0.6
*r* ^*^		0.135	0.135	0.138	0.120	0.132
*P*-value		0.455	0.455	0.445	0.506	0.464

* Spearman correlation coefficient between dietary sialic acid intake and sialic acid content and concentration in human milk.

a P < 0.05, compared with 90th Dietary Total SA intake.

The average milk yield of lactating mothers on the 3rd, 8th, 30th, and 90th day after delivery was 100, 500, 650, and 900 ml, respectively, while the total amount of sialic acid secreted by lactating mothers was 167.07 ± 9.45, 636.09 ± 64.37, 352.07 ± 35.88, and 267.89 ± 18.7 mg, respectively. And the result was estimated according to the average milk yield and the concentration of sialic acid in milk. The amount of sialic acid consumed from the diet on the 2nd, 7th, 30th and 90th days was 106.06 ± 7.51, 127.64 ± 8.61, 120.34 ± 10.21, and 95.40 ± 6.34 mg, respectively. Statistical analysis showed that there were significant differences in dietary sialic acid intake among lactating mothers at different time points, and the dietary sialic acid intake on the 7th day and 30th day was significantly higher than that on the 90th day (*P* < 0.05). In addition, the total amount of sialic acid in breast milk at different postpartum time points was higher than that of dietary intake.

The results of the normality test of dietary sialic acid intake of lactating mothers and sialic acid content in milk at four different time points showed that the data were normally distributed (*P* > 0.05), while the data of total sialic acid secretion and content in each milk form were not normally distributed (*P* < 0.05). Therefore, Spearman correlation was used to analyze the correlation between dietary sialic acid and that in milk; no significant correlation was observed (*P* > 0.05). Similarly, there was no statistically significant correlation between the total and other forms of sialic acid and the dietary sialic acid intake (*P* > 0.05) during different lactation periods.

## Discussion

In this study, the sialic acid content in human milk was inversely correlated with postpartum days and gradually decreased with the prolonged lactation period. There was a great difference in the concentration at different times. This trend of sialic acid content in milk results from long-term human evolution, which is adapted to the characteristics of infant growth and development. Compared with other foods, the sialic acid content in human milk is very high (9), especially in colostrum. Sialic acid is mainly synthesized in the liver; GNE (UDP-GlcNAc 2-epimerase) is a key enzyme in regulating sialic acid biosynthesis (10). In the neonatal period, this enzyme cannot meet the needs of rapid brain development due to the immature liver and limited ability to regulate sialic acid synthesis. However, the high sialic acid content in early colostrum can be used as an exogenous supplement to make up for the deficiency of endogenous synthesis in newborns. With continued lactation, the liver function of infants gradually improves, and the ability to synthesize sialic acid gradually improves, which can meet the baby's needs to a greater extent and reduces exogenous dependence. At this time, the decreasing trend of sialic acid content in milk conforms to the changes in the baby's needs. Therefore, the different sialic acid requirements of babies in different lactation periods may lead to different levels of sialic acid in milk, and the dynamic changes of sialic acid in human milk may be determined by different lactation needs.

In addition to the fluctuation of sialic acid content with time, there are also great differences among different individuals, confirmed by many earlier studies (11, 12). In this study, the total sialic acid content of different individuals fluctuated greatly at four different lactation stages. The maximum sialic acid content of 1-month mature milk is about 6.8 times the minimum. The lipid content of human milk is the most variable, and the lipid content of different individuals varies by about 7 times (0.4–2.8 g/100 ml) in 1-month mature milk (13, 14), and the content of sialic acid varies among individuals. Thus, sialic acid is one of the most obvious variable components of human milk. The reason for variation in the sialic acid content of human milk of different individuals is unclear. It may be due to genetic differences that cause different sialic acid synthesis capabilities; that is, the enzymatic activity of synthesizing sialic acid in different lactating mothers is different, and the amount of synthesized sialic acid may also be different. It is also possible that the stimulation of environmental factors leads to different utilization rates of sialic acid *in vivo*.

From the existing form of sialic acid in human milk, it is mainly in the form of oligosaccharide-bound. The sialic acid concentration in all fractions decreased significantly with a longer lactation duration. However, the proportion of sialic acid composition in each fraction did not differ significantly between lactation periods. Oligosaccharide-bound sialic acid always occupies a high proportion in human milk, which may be determined by the content of oligosaccharides in human milk. The main sialylated oligosaccharides in human milk were 3′-SL (3′-siaiyllyatose) and 6′-SL (6′-siaiyllyatose). The concentration of 6′-SL was the highest in colostrum (250–1,300 mg/L) and then decreased gradually, while the concentration of 3′-SL was relatively stable (76–300 mg/L) (15). Studies have shown that human milk contains an appreciable amount of oligosaccharides, with an average content of 12.9 g/L (16), the third-largest component of human milk solids. There are no less than 200 kinds of oligosaccharides in human milk (17). This creates good conditions for combining sialic acid and oligosaccharides in human milk. Oligosaccharides in human milk can not only resist some enterovirus and bacterial infections (18) and participate in the development of a healthy intestinal environment but also stabilize sialic acid in milk and avoid its related enzymatic hydrolysis, and even alter the sialic acid metabolism pathway to improve the half life. According to the above, oligosaccharides are very important components in breast milk. After all kinds of oligosaccharides are sialylated, they endow breast milk with unique functional advantages, which are not available in ordinary formula milk and cow milk. In this study, we determined the total concentration of all kinds of oligosaccharide-bound sialic acid after hydrolysis, without subdividing specific sialic acid containing oligosaccharides. Further detection of the type and concentration of oligosaccharide-bound sialic acid may be more specific to show the distribution of sialic acid in breast milk and its relationship with diet.

During lactation, especially within 1 month after delivery, mothers pay more attention to various nutrients intake; thus, food intake is adequate, and the food structure is reasonable. The effects of the dietary intake of lactating mothers on the composition of human milk are complex and diverse, and different nutrients may be affected differently. Studies have shown (19, 20) that the content of macronutrients in human milk is almost not affected by the intake of macronutrients in the maternal diet. However, specific fatty acids that form lipid parts are susceptible to being affected by lactating mothers' diets (21).

The correlation of Neu5Ac concentration in human milk with the dietary intake of lactating women was also not found in our current study. Besides, there were no significant correlations between the contents of different forms of sialic acid in human milk and the dietary intake of lactating women (*P* > 0.05). This suggests that the content of sialic acid in milk may not be affected by the mother's diet. Human milk nutrients generally come from three sources: some may be synthesized by breast cells, some from the mother's diet, and some from the mother's nutritional reserves (22).

From the previous results, the amount of sialic acid secreted by mothers through milk at different stages of lactation is higher than their dietary intake, especially in the transition milk, where the total sialic acid secretion in milk is about five times higher than the dietary intake. It can be inferred that the sialic acid in human milk mainly comes from self-synthesis or reserve. When the total content of non-dietary sialic acid is significantly higher than dietary intake, the correlation between sialic acid content in human milk and dietary sialic acid intake may appear weak or directly covered.

In addition, the amount of sialic acid taken by lactating mothers through their diet at different times has little change. However, the content of sialic acid in milk shows a downward trend with the prolonged lactation period, which shows that sialic acid in milk may depend more on the physiological needs of lactating mothers than on their dietary intake. Certain self-regulatory mechanisms in the lactating mother's body might be responsible, and dietary sialic acid intake fluctuations are buffered by this mechanism, thus maintaining a relatively balanced physiological state of sialic acid content in milk, which does not change with dietary sialic acid intake. Perhaps when the content of sialic acid in human milk is low or relatively deficient, dietary sialic acid intake might play a supplementary role, and the correlation between dietary and sialic acid in human milk can be seen more clearly. When the sialic acid content in human milk has been at a high level in the corresponding stage, in a plateau phase, excessive dietary sialic acid intake does not affect the overall level of sialic acid in human milk; therefore, there is no significant correlation between diet and sialic acid in milk. The dietary investigation in this study was 24 h before milk collection; this may not rapidly affect the change of sialic acid content in human milk. Dietary intake of sialic acid was mainly in bound form, and the dietary bound form of sialic acid is better absorbed and utilized by body tissues. However, absorption into the blood and secretion into human milk may take time.

Finally, the study considered that the analysis of intake vs. secretion compared to intake vs. concentration, which may better reveal the correlation. Therefore, we not only explored the relationship between dietary sialic acid intake and secretory concentration, but also further analyzed the correlation between total sialic acid intake and secretion. Since the amount of milk secreted by an individual is uneven and fluctuates greatly, it is difficult to get a stable value of milk production. So we take the average value of standard milk from a large sample as a reference to calculate the total amount and make a relevant analysis. However, even when the amount of milk at different stages was taken into account, it failed to further suggest a correlation between diet and sialic acid in milk, but rather verified to some extent that the two were not correlated.

This study draws the following conclusions: The total sialic acid concentrations were highest in colostrum and decreased with the lactation period; Sialic acid in human milk is mostly oligosaccharide-bound, some protein-bound, and a little in the free form. The distribution of sialic acid in human milk is unchanged at different stages of lactation. In addition, the sialic acid in human milk is higher than dietary intake, without any correlation between them; the content in human milk mainly depends on the self-regulation of physiological needs.

## Data availability statement

The raw data supporting the conclusions of this article will be made available by the authors, without undue reservation.

## Ethics statement

The studies involving human participants were reviewed and approved by the Medical Ethics Committee. The patients/participants provided their written informed consent to participate in this study.

## Author contributions

QX and YX: conceptualization, software, and writing—original draft preparation. XW, JH, HL, MZ, and YZ: investigation. XC, DG, and HL: resources and supervision. QX, YX, and WZ: data curation. QX: writing review and editing. XC, DG, XW, and HL: project administration and funding acquisition. All authors have read and agreed to the published version of the manuscript.

## Funding

This research was supported by a Grant from the Incubation Project of Xiamen Hospital, Zhongshan Hospital affiliated to Fudan University (2020ZSXMYS24) to XW.

## Conflict of interest

The authors declare that the research was conducted in the absence of any commercial or financial relationships that could be construed as a potential conflict of interest.

## Publisher's note

All claims expressed in this article are solely those of the authors and do not necessarily represent those of their affiliated organizations, or those of the publisher, the editors and the reviewers. Any product that may be evaluated in this article, or claim that may be made by its manufacturer, is not guaranteed or endorsed by the publisher.
